# Diacylated lipopeptide from *Mycoplasma synoviae* mediates TLR15 induced innate immune responses

**DOI:** 10.1186/1297-9716-44-99

**Published:** 2013-10-17

**Authors:** Irena Oven, Katarina Resman Rus, Daliborka Dušanić, Dušan Benčina, Calvin L Keeler, Mojca Narat

**Affiliations:** 1 Department of Animal Science, Biotechnical Faculty, University of Ljubljana, Groblje 3, SI-1230 Domžale, Slovenia; 2 Department of Animal and Food Sciences, College of Agriculture and Natural Resources, University of Delaware, 040 Townsend Hall, Newark, DE 19717-1303, USA

## Abstract

Avian-specific toll like receptor 15 (TLR15) is functionally equivalent to a group of TLR2 family proteins that the mammalian innate immune system utilizes to recognize a broad spectrum of microbe-associated molecular patterns, including bacterial lipoproteins. In this study we examined the role of chicken TLR2 family members in the innate immune response to the avian pathogenic bacterium, *Mycoplasma synoviae*. We found that *Mycoplasma synoviae,* and specifically the N-terminal diacylated lipopeptide (MDLP) representing the amino-terminal portion of its mature haemagglutinin protein, significantly induces the expression of *TLR15*, but not *TLR1* and *TLR2* in chicken macrophages and chondrocytes. TLR15 activation is specific and depends on diacylation of the lipopeptide. Activation of TLR15 after stimulation with *Mycoplasma synoviae* and MDLP triggers an increase in the expression of transcription factor nuclear factor kappa B and nitric oxide production. Moreover, transfection of avian macrophage cells with small interfering RNA reduces the expression of *TLR15* after stimulation with MDLP. This leads to decreased activation of the innate immune response, as measured by nitric oxide production. Additionally, pretreatment of cells with neutralizing anti-TLR15 antibody results in a notable attenuation of MDLP-driven release of nitric oxide. This positive correlation may constitute a mechanism for stimulating the innate immune response against avian mycoplasmas in chicken cells via TLR15.

## Introduction

Mycoplasmas are the smallest self-replicating organisms, and are distinguished from other bacteria by their small size and total lack of a cell wall. As obligate parasites they usually exhibit strict host and tissue specificity. Mycoplasmas have been shown to interact with the host’s immune system on many levels, which includes modulating the host immune system and stimulating an inflammatory response. These abilities enable mycoplasmas to establish a chronic, persistent infection (reviewed in [1]). In poultry the most pathogenic species are *Mycoplasma synoviae* and *Mycoplasma gallisepticum*. *Mycoplasma synoviae* most frequently colonizes the upper respiratory tract, causing subclinical infections, although this condition can also lead to the development of systemic infection and/or infectious synovitis in chickens and turkeys [2,3]. In the absence of a cell wall, the majority of the mycoplasma surface antigens are lipoproteins. In the avian pathogens *Mycoplasma gallisepticum* and *Mycoplasma synoviae* an abundantly expressed variable lipoprotein haemagglutinin (VlhA) is believed to play a major role in pathogenesis of the disease by mediating adherence and immune evasion [4]. VlhA is post-translationaly cleaved into 2 proteins, the amino terminal lipoprotein portion MSPB and the more antigenically variable C terminal haemagglutinin MSPA. In phenotypically distinct *Mycoplasma synoviae* populations truncated forms of MSPB (tMSPB) also occur [3,5,6]. Both MSPB and tMSPB contain an amino terminal proline rich region [5], which has been shown to induce strong local and systemic antibody responses in infectious synovitis [3] and the production of proinflammatory cytokines and other effector molecules [7], although the mechanisms underlying this response are still not clear. Other *Mycoplasma* lipoproteins and lipopeptides have also been found to be subject to similar post-translational modifications. One of these is the macrophage stimulatory lipopeptide MALP-2 from *Mycoplasma fermentas*, which is derived from a 43 kDa surface lipoprotein that is post-translationally cleaved into the mature 14-residue long N-terminal lipopeptide MALP-2 [8].

Bacterial lipoproteins, including mycoplasmal lipoproteins, belong to a group of pathogen associated molecular patterns (PAMPs) which interact with Toll like receptors (TLRs) present on host cells [9]. In humans, TLR1, TLR2 and TLR6 are implicated in the recognition of bacterial lipoproteins, where TLR2 dimerizes with either TLR1 or TLR6 to enhance the recognition of lipoproteins and elicit the cellular cytokine response through the activation of nuclear factor kappa B (NF-κB ) and the mitogen activated protein kinase signaling cascade [10].

In birds, 10 avian TLRs have been described so far, of which TLR2a, TLR2b, TLR3, TLR4, TLR5, and TLR7 are orthologs to those found in humans. Additionally, avian TLR21, which is absent from mammalian genome, is the functional homologue of mammalian TLR9, recognizing CpG DNA. Besides TLR2a and TLR2b, members of the avian TLR2 group also include two TLR1-like proteins (TLR1La and TLR1Lb), and TLR15, which appears to be unique to avian and reptile species [11]. Orthologs of mammalian TLR6 and TLR10 have not been found in birds (reviewed in [12]). Several studies report that both types of chTLR1L can interact with both types of chTLR2 in different combinations in response to bacterial lipoproteins, although the findings of these studies are somewhat contradictory [13-15]. TLR15 has so far been identified only in avian and reptile species [11]. Its induction was first observed in the cecum of chickens infected with *Salmonella enterica*[16]. Later studies have also reported the induction of chTLR15 after stimulation of chicken fibroblasts and heterophils by heat-killed Gram-negative and Gram-positive bacteria, but not by any of the other tested TLR agonist [17-20]. A more recent study reported the induction of *TLR15* mRNA expression after stimulation with CpG-oligonucleotide (CpG-ODN), tripalmitoylated lipopeptide (PAM3CSK4) and lipopolysaccharide (LPS) [21], whereas another study suggested a novel mechanism of activation, where TLR15 is activated through its cleavage by microbial proteases [22]. A third recent study showed that yeast lysates can induce the TLR15-dependent activation of NF-κB expression, however, the exact agonist was not identified [11]. Nevertheless, the fact that TLR15 induction appears to be unique to the avian species and is molecularly distinct from other known TLRs, suggests a specific and unique role in defense against avian pathogens [18].

In this study we report a novel ligand for TLR15, a diacylated lipopeptide derived from *Mycoplasma synoviae*, a common chicken pathogen. We used a synthetic diacylated lipopetide (MDLP) based on the N-terminal sequence of MSPB, and its diacyl modification was chosen by anology to other *Mycoplasma*-derived lipopeptides (eg. MALP-2, FSL-1). Its non acylated peptide analog (NAP) was also synthesized. We found that MDLP was capable of inducing *TLR15* expression, which led to NF-κB activation and nitric oxide production.

## Materials and methods

### Reagents and chemicals

Unless otherwise noted, reagents and chemicals were purchased from Sigma–Aldrich Corp., St. Louis, USA.

### *Mycoplasma synoviae* culture

*Mycoplasma synoviae* strains WVU 1853 and ULB 01/P4 were grown at 37 °C on modified Frey’s medium containing 12% porcine serum (Life Technologies Inc., Gaithersburg, USA) and 0.1 g of NAD per liter of broth medium (Merck & Co. Inc., Whitehouse Station, USA), but without addition of thallium acetate [23].

### MSPB lipoprotein isolation and lipopetide / peptide determination

MSPB lipoprotein was isolated from *Mycoplasma synoviae* strain ULB 01/P4 as previously described [7]. The amino acid sequence of the N-terminal region of MSPB proteins of type strain WVU1853 and strain ULB 01/P4 were predicted previously [5] from the 5′-end of the *vlhA* gene sequence. The proposed N-terminal amino acid sequence (CGDQTPAPEPTPGNPNTDNPQNPN) was the same in both strains. Based on this sequence, the 14 amino acid NAP peptide (CGDQTPAPEPTPGN) was synthesized, as well as the corresponding lipopeptide, MDLP, containing an S-(2,3-bispalmitoyloxypropyl) N-terminal modification (Pam_2_CGDQTPAPEPTPGN) (both EMC microcollections GmbH, Tuebingen, Germany), which mimics the putative diacyl lipid moiety found in mycoplasma lipoproteins [8].

### Cell cultures

The chicken macrophage cell line HD11 was cultured in RPMI medium, supplemented with 8% fetal bovine serum (FBS) and 2% chicken serum, at 37 °C in a 5% CO_2_ atmosphere. Monocyte-derived macrophages (MDM) were prepared by Histopaque®-1077 density gradient centrifugation of chicken blood as described previously [7,24] and plated in RPMI 1640 medium, supplemented with 10% FBS (Hyclone, USA), 100 U/mL penicillin, and 100 U/mL streptomycin, at 41 °C in a 5% CO_2_ atmosphere.

Primary chicken chondrocytes (CCH) were isolated as previously described [25]. CCH were cultivated up to six passages in DMEM medium supplemented with 7.5% FBS and 2.5% chicken serum. Cells were incubated at 37 °C in a 5% CO_2_ atmosphere.

### Macrophage exposure to *Mycoplasma synoviae*, Pam_2_CGDQTPAPEPTPGN lipopeptide (MDLP) and CGDQTPAPEPTPGN peptide (NAP)

The number of *Mycoplasma synoviae* cells per macrophage (HD11) was adjusted using the colony forming units (CFU) technique, as previously described [24]. HD11 cells were exposed to bacteria by replacing their growth medium with medium containing bacteria at a multiplicity of approximately 100 viable *Mycoplasma synoviae* cells per macrophage. HD11, CCH and MDM cells were exposed to 1 μM of MDLP or 1 μM NAP. Non-exposed cells were used as negative controls. Cell cultures exposed to different agents were incubated at 37 °C (for HD11 and CCH) or 41 °C (for MDM) and 5% CO_2_ before harvesting cells for RNA. At 1, 6, or 24 h of exposure, growth medium was aspirated from plates, RLT lysis buffer (Qiagen Corp., Valencia, USA) was added and cells were harvested with a rubber policeman.

### Quantitative real-time RT-PCR

To confirm and validate gene expression changes quantitative real-time RT-PCR (RT-qPCR) was performed on: ch*TLR15*, ch*TLR1*, ch*TLR2*, *iNOS* and *NF-κB* using *GAPDH* as a housekeeping control gene (Table 1). RT-qPCR primers were designed using PrimerQuest^SM^ (Integrated DNA Technologies, Leuven, Belgium) and checked for specificity in silico with PrimerBlast (NCBI). RNA was isolated from cells using the RNAeasy mini kit following on- column DNase-I digestion in accordance with the manufacturer’s protocol (Qiagen). cDNA was reverse transcribed using the High-Capacity cDNA Reverse Transcription Kit (Applied Biosystems, Foster City, USA). RT-qPCR assays were performed using the 2× FastStart Universal SYBR Green Master Mix (Rox) (Roche Diagnostics GmbH, Mannheim, Germany). Quantitative PCR was performed for each sample in triplicate on an Mx3000p QPCR System (Agilent Technologies – Stratagene, Santa Clara, USA). The three-step amplification procedure was performed in a 20 μL reaction volume containing 300 nM of each primer and 150 pg of cDNA. Reaction conditions were set to 10 min at 95 °C (first segment, one cycle), 15 s at 95 °C and 1 min at the Tm of the specific primer pair (second segment, 40 cycles) followed by one cycle with 15 s at 95 °C, 30 s at the designated Tm and 15 s at 95 °C (dissociation curve segment). Data were analyzed using MxPro 4.0 Software (Agilent Technologies). Gene expression values of non-infected or non-treated cells were used for gene expression calibration. Appropriate controls (no template and no reverse transcription control) were also performed in each run. At least three independent experiments were performed to collect RNA for RT-qPCR. Relative gene expression was assayed in each experiment and experimental condition separately. Normalized relative quantities were calculated using the efficiency corrected 2^-(ΔΔCt)^ method [26]. The effect of intra-assay variation on the statistical significance of the results was reduced by log transformation of normalized relative quantities, mean centering and autoscaling by the method of Willems et al. [27].

**Table 1:** Oligonucleotides used as primers in RT-qPCR analysis of gene expression in chicken cells.

**Gene symbol**	**RefSeq mRNA number**	**Forward primer**	**Reverse primer**
*GAPDH*	NM_204305.1	ATCGTCAAGGCTGAGAACGGG	ATCTGCCCATTTGATGTTGCT
*TLR15*	NM_001037835.1	AACCTGGTGCATTTGAGAACCTGC	TTTCAGGTGAGGTGCAAGACCAGA
*TLR1*	NM_001007488.3	AGCTGTGTCAGCATGAGAGGAACT	AGTTGGGCGACAACACAAAGATGG
*TLR2*	NM_001161650.1	AGAACGACTCCAACTGGGTGGAAA	AGAGCGTCTTGTGGCTCTTCTCAA
	NM_204278.1		
*iNOS*	NM_204961.1	GCATTCTTATTGGCCCAGGA	CATAGAGACGCTGCTGCCAG
*NF-κB*	NM_205134.1	AGGACTTAAAATGGCAGGAGA	GCTGTTCGTAGTGGTAAGTCT

### Western blotting

1 × 10^6^ HD11 cells were cultured in RPMI containing 8% FBS and 2% chicken serum overnight and were then stimulated with 1 μM MDLP or NAP for 24 h. Cell lysates were prepared in RIPA buffer. Protein content was determined and equal amounts of lysates were fractionated by 10% SDS-PAGE and electrotransferred to polyvinylidene difluoride membranes. Rabbit polyclonal anti-TLR15 antibody (Imgenex, San Diego, USA) and anti -actin 2Q1055 antibody (Abcam, Cambridge, UK) were used for the detection of proteins. Detection was by an enhanced chemiluminescence system (Amersham, Freiburg, Germany).

### Nitric oxide assay

Nitric oxide (NO), induced in HD11 cells by DLP, was estimated, with some modifications, as previously described [7]. Briefly, HD11 cells were seeded into 24-well plates (2 × 10^5^ cells / well) and NAP or MDLP was added to a final concentration of 0.5, 1 or 1.5 μM. As a positive control HD11 cells were incubated in the presence of 0.1 μg/mL E. coli O127:B8 LPS. Cell culture supernatants were sampled after 6 and 24 h of incubation at 37 °C, in 5% CO_2_. Control supernatants were sampled after 24 h. Quantification of nitric oxide was performed using the Griess Reagent System (Promega GmbH, Mannheim, Germany), according to the manufacturer’s instructions.

### RNA silencing

Silencing of TLR15 was performed using the Screening DsiRNA TriFECTa Kit (cat.# GGC.RNAI.N001037835.12, Integrated DNA Technologies). A universal negative control siRNA (NC siRNA) not homologous to any known transcript in the vertebrate transcriptome, was used to normalize relative gene inhibition of the target gene. A positive control siRNA against *HPRT*-1 was used to assess transfection and knockdown efficiency. Twenty-four h before transfection, 8 × 10^4^ HD11 cells / well were seeded onto a 24-well plate to reach 40-50% confluence. These cells were transfected with a cocktail of three TLR15 siRNAs (150 nM total) or a nonsense negative control (NC siRNA), using the chemical transfection reagent X-treme GENE siRNA Transfection reagent (Roche). Transfection was performed according to the manufacturer’s instructions. Briefly, siRNAs were diluted in 50 μL Opti-MEM medium and mixed gently. Four μL of X-tremeGENE was diluted in 50 μL Opti-MEM medium and combined with the diluted RNAi duplex, incubated for 20 min at room temperature, then added to each well containing cells to give a final volume of 500 μL and a final siRNA concentration of 150 nM. The conditions for siRNA transfection were optimized by adjusting different siRNA concentrations (10, 50, and 150 nM) and different transfection methods (reverse and forward) using Tye 563™ Fluorescent Control (Integrated DNA Technologies). Transfection efficiency was evaluated under a fluorescent microscope. Eight hours after transfection, HD11 cells were treated with 1 μM MDLP or NAP for 12 h, and RNA isolated from cells or NO was measured in the supernatant as described above.

### Antibody neutralization assay

HD11 cells (5 × 10^4^ cells / well) were seeded onto 96-well culture plates and pre-treated with 2.5 μL of rabbit polyclonal anti-TLR15 antibodies (Imgenex), 2.5 μL of normal rabbit serum (1:1 in glycerol) or PBS for 1 h at 37 °C in 5% CO_2_. Following the 1 h incubation, 1 μM MDLP or NAP were applied in the continuing presence of neutralizing antibodies, and the cells were further incubated overnight in the same conditions. NO from cell supernatants was determined as described above.

### Statistical analysis

Statistical analysis was performed for selected experiments to determine the confidence limit at which two measurements were statistically different. A Student *t*-test (*p* < 0.05) was applied to each dataset in SigmaPlot program, and *p*-values were obtained and are reported in the figure legends.

## Results

### Expression of TLR15, TLR1 and TLR2 genes after infection of HD11 cells with *Mycoplasma synoviae*

**Figure 1:**
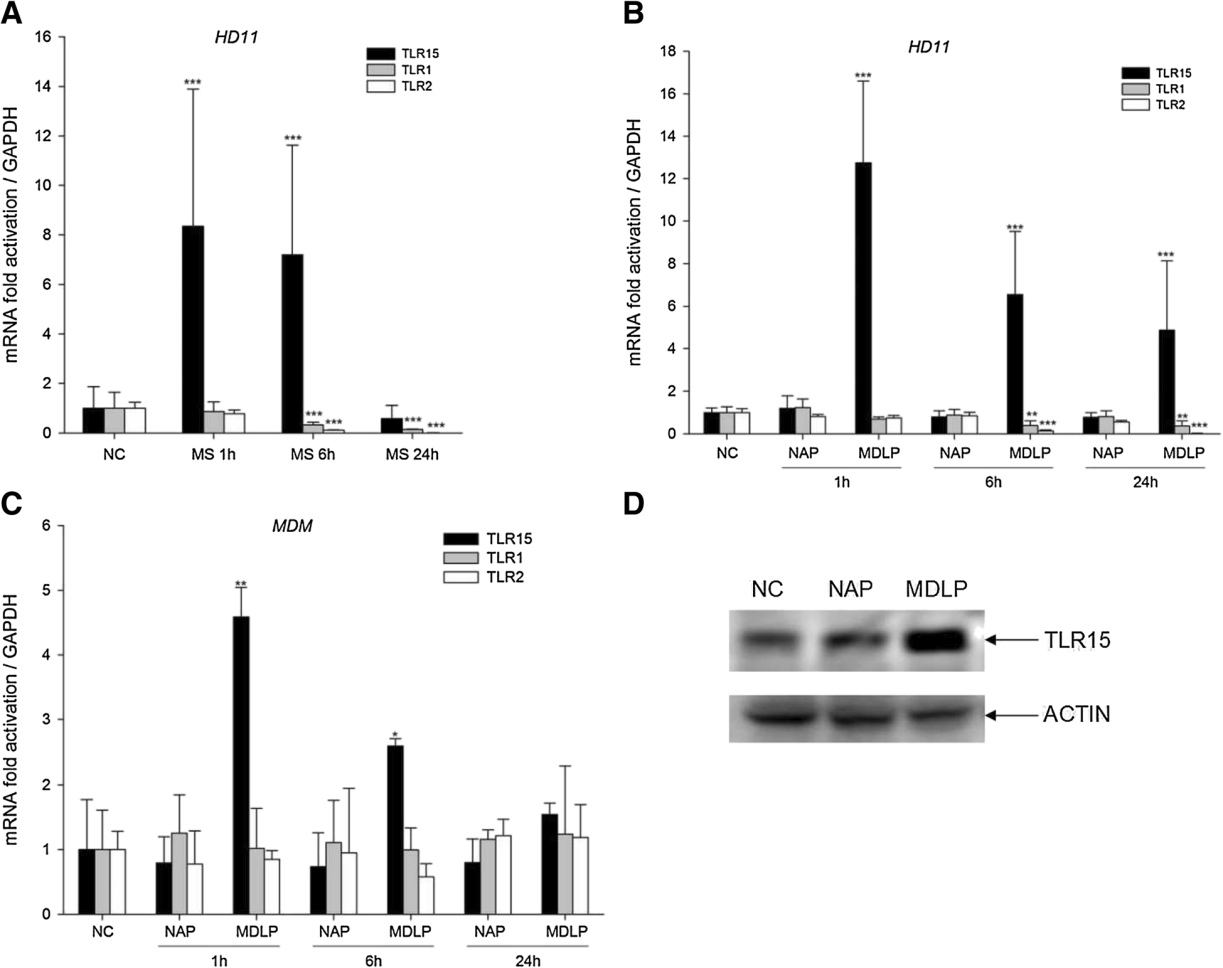
***Mycoplasma synoviae *and its diacylated lipopeptide induce expression of TLR15 in chicken macrophages. A)** Expression of *TLR15*, *TLR1* and *TLR2* mRNA after infection of HD11 cells with *Mycoplasma synoviae*. HD11 cells were infected with live *Mycoplasma synoviae* (MS) for 1 h, 6 h or 24 h and mRNA expression was analyzed by RT-qPCR. NC represents non-infected cells. Bars show mean ± S.E. (*n* = 4); ***, *p* < 0.001 compared to non-infected cells. **B)** Expression of *TLR15*, *TLR1* and *TLR2* mRNA after treatment of HD11 cells with diacylated lipopeptide or its non-acylated analog. HD11 cells were treated with 1 μM non-acylated peptide (NAP) or diacylated lipopeptide (MDLP) for 1 h, 6 h or 24 h and mRNA expression was analyzed by RT-qPCR. NC represents non-treated cells. Bars show mean ± S.E. (*n* = 4); **, *p* < 0.01 and ***, *p* < 0.001 compared to NAP-treated cells at each time point for selected gene. **C)** Expression of *TLR15*, *TLR1* and *TLR2* mRNA after treatment of MDM cells with diacylated lipopeptide or its non-acylated analog. MDM cells were treated with 1 μM non-acylated peptide (NAP) or diacylated lipopeptide (MDLP) for 1 h, 6 h or 24 h and mRNA expression was analyzed by RT-qPCR. NC represents non-treated cells. Bars show mean ± S.E. (*n* = 3); *, *p* < 0.05 and **, *p* < 0.01 compared to NAP-treated cells at each time point for selected gene. **D)** Expression levels of TLR15 and β-actin, as determined by Western blotting. HD11 cells were treated with NAP or MDLP for 24 h and TLR15 protein levels were determined. Levels of endogenous β-actin were determined by Western blotting to validate input for each sample.

We first sought to determine which chicken TLR2 family members are involved in the immune response to *Mycoplasma synoviae* infection of macrophages. Therefore, we analyzed the expression of *TLR15*, *TLR1* and *TLR2* genes after infection of HD11 macrophages with live *Mycoplasma synoviae* for 1, 6 or 24 h. For the analysis of *TLR1* and *TLR2* mRNA expression we used primers that recognized both forms of each chTLR receptors. As shown in Figure 1A, infection of HD11 cells with *Mycoplasma synoviae* significantly induces the expression of *TLR15* (approximately 8-fold, *p* < 0.001) after 1 h and 6 h, whereas after 24 h the expression of *TLR15* was not up-regulated compared with non-infected cells. Interestingly, the expression of *TLR1* and *TLR2* was not up-regulated; moreover, they were significantly down-regulated after 6 h and 24 h (*p* < 0.001) (Figure 1A).

It was previously shown that the N-terminal lipoprotein fraction of VlhA is responsible for the induction of a strong immune response [7]. Based on the N-terminal sequence of the MSPB protein, and by analogy to MALP-2 and FSL-1, we synthesized the Pam_2_CGDQTPAPEPTPGN lipopeptide, termed MDLP, and its non-acylated peptide analog, NAP. We analyzed the transcription levels of the *TLR15*, *TLR1* and *TLR2* genes in HD11 cells after treating them with either 1 μM MDLP or NAP for 1 h, 6 h or 24 h. As shown in Figure 1B MDLP significantly induces the expression of *TLR15* compared with non-treated cells and NAP-treated cells. The expression of *TLR15* was highest after 1 h of exposure (13-fold compared with non-treated cells, *p* < 0.001). The expression of *TLR15* was not up-regulated when the cells were treated with the non-modified peptide (Figure 1B, NAP). The expression of *TLR15* was still up-regulated after 6 h and 24 h (6.5-fold and 5-fold, respectively, *p* < 0.001). Interestingly, as with the *Mycoplasma synoviae* infection experiment, the mRNA expressions of *TLR1* and *TLR2* were significantly down-regulated after the treatment of the cells with MDLP (*p* < 0.01), whereas when treated with NAP, the expression remained the same compared to non-treated cells (Figure 1B, TLR1 and TLR2, respectively).

Since HD11 cells are a virally transformed macrophage cell line, we wanted to confirm that this cell line is a suitable model for our experiments by analyzing the expression of *TLR15*, *TLR1* and *TLR2* genes in primary macrophages. Therefore, we isolated monocyte derived macrophages from chicken blood and treated them with 1 μM of MDLP or NAP for 1 h, 6 h or 24 h. At each time point expression of the *TLR15*, *TLR1* and *TLR2* genes was analyzed. As shown in Figure 1C MDLP significantly induces the expression of *TLR15* compared with non-treated cells and NAP-treated cells. The expression of *TLR15* was highest after 1 h of exposure (4.6-fold compared with non-treated cells, *p* = 0.003). The expression of *TLR15* was not up-regulated when the cells were treated with the non-modified peptide (Figure 1C, NAP). At longer treatment times (6 h and 24 h) the expression of *TLR15* was still induced although the level was not statistically significant (2.6-fold and 1.5-fold, *p* = 0.025 and *p* = 0.139, respectively).

To confirm that the expression of TLR15 was induced not only at the mRNA level, but also at the protein level, we performed a western blot analysis in HD11 cells after 24 h of exposure to MDLP or NAP. Changes in protein levels were consistent with the mRNA data, showing that NAP did not affect the expression of TLR15 protein, whereas treatment with MDLP induced up-regulation of TLR15 protein expression (Figure 1D).

**Figure 2:**
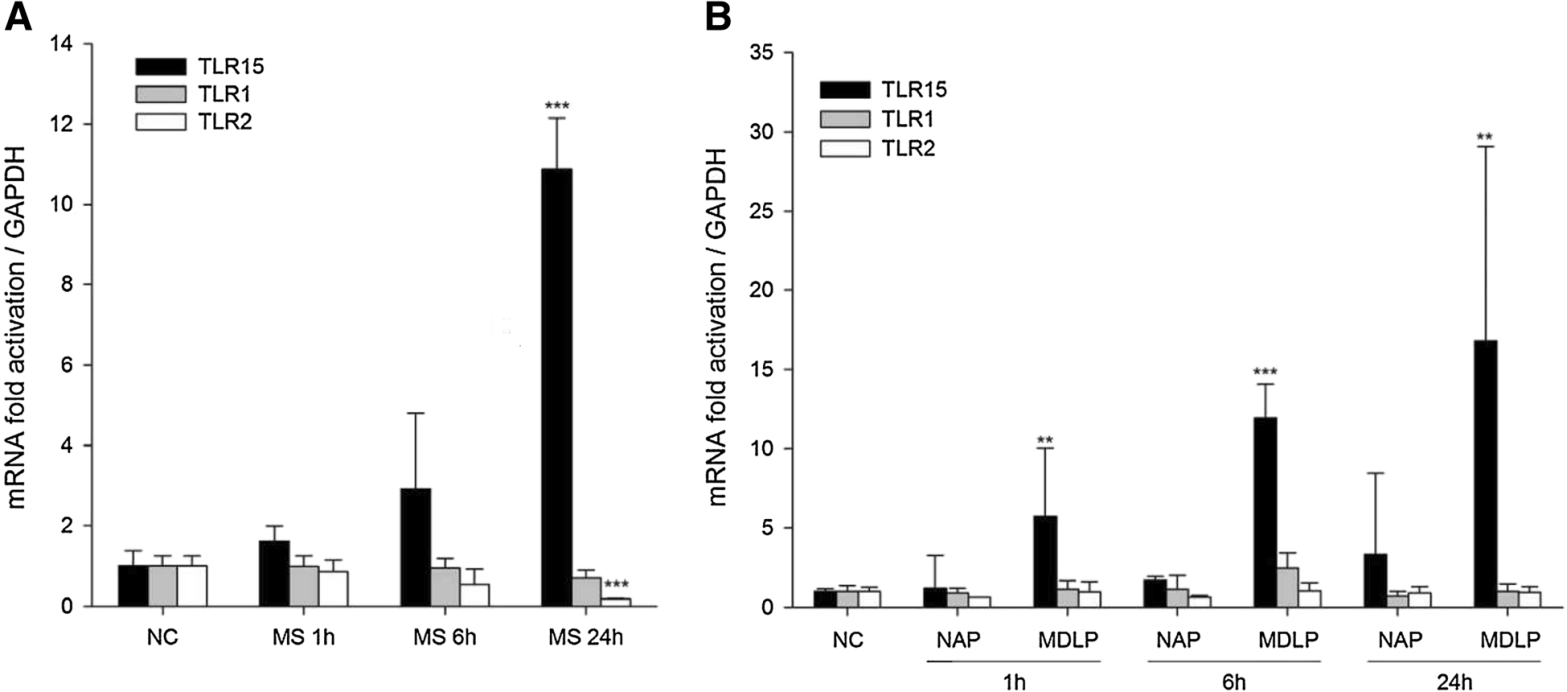
***Mycoplasma synoviae *and its diacylated lipopeptide induce expression of TLR15 in primary chicken chondrocytes. A)** Expression of *TLR15*, *TLR1* and *TLR2* mRNA after infection of cells with *Mycoplasma synoviae*. Primary chicken chondrocytes were infected with live *Mycoplasma synoviae* (MS) for 1 h, 6 h or 24 h and mRNA expression was analyzed by RT-qPCR. NC represents non-infected cells. Bars show mean ± S.E. (*n* = 4); ***, *p* < 0.001 compared to non-infected cells. **B)** Expression of *TLR15*, *TLR1* and *TLR2* mRNA after treatment of cells with diacylated lipopeptide or its non-acylated analog. Primary chicken chondrocytes were treated with 1 μM non-acylated peptide (NAP) or diacylated lipopeptide (MDLP) for 1 h, 6 h or 24 h and mRNA expression was analyzed by RT-qPCR. NC represents non-treated cells. Bars show mean ± S.E. (*n* = 4); **, *p* < 0.01 and ***, *p* < 0.001 compared to NAP-treated cells at each time point for selected gene.

*Mycoplasma synoviae* has been shown to interact with non-immune cells, and is often isolated from the joints of chickens showing signs of infectious synovitis. To investigate if *Mycoplasma synoviae* can modify the expression of chicken TLR2-family genes in non-immune cells, we infected primary chicken chondrocyte cells (CCH) with *Mycoplasma synoviae* for 1 h, 6 h and 24 h, and analyzed the expression of *TLR15*, *TLR1* and *TLR2* genes. In contrast to the response in the immune cells, the expression of *TLR15* was the highest after 24 h of infection (11-fold, *p* < 0.001), whereas *TLR1* and *TLR2* expression remained unchanged or was even slightly down-regulated, correlating to the results observed in chicken macrophages (Figure 2A). We found similar results when we treated CCH cells with MDLP or NAP. MDLP induced *TLR15* expression in CCH after 1 h (Figure 2B, 6-fold, *p* = 0.005), and the expression of the *TLR15* was further increased after 6 h and 24 h (Figure 2B, 12-fold and 17-fold, *p* < 0.001 and *p* = 0.002, respectively). NAP did not change the expression of any of the analyzed TLRs compared with non-infected cells (Figure 2B).

### Increased TLR15 expression is associated with increased NF-κB, iNOS message and nitric oxide production

**Figure 3:**
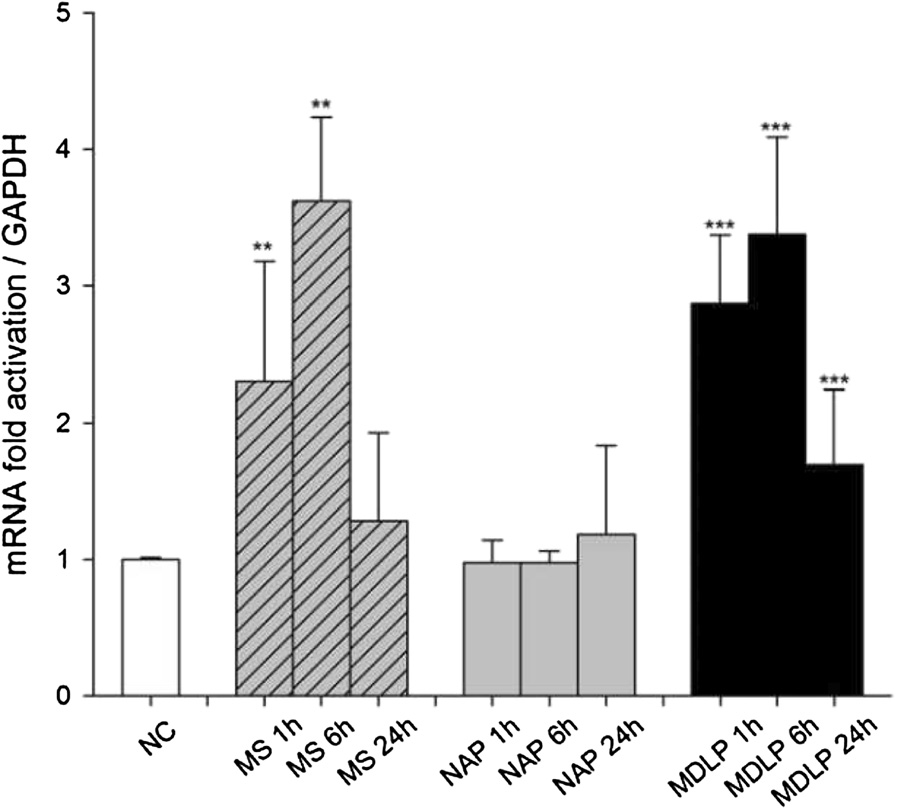
***Mycoplasma synoviae *and its diacylated lipopeptide induce transcription of *NF-κB *in HD11 macrophages.** HD11 cells were infected with *Mycoplasma synoviae* (MS) or treated with 1 μM non-acylated peptide (NAP) or diacylated lipopeptide (MDLP) for 1 h, 6 h or 24 h and mRNA expression was analyzed by RT-qPCR. NC represents non-infected cells. Bars show mean ± S.E. (*n* = 6); *, *p* < 0.05 and ***, *p* < 0.001 compared to non-treated cells for *Mycoplasma synoviae* or NAP-treated cells at each time point, respectively.

To further investigate the mechanism of the TLR15 mediated innate immune response, we measured *NF-κB* mRNA expression following infection of HD11 cells with viable *Mycoplasma synoviae* or treatment with MDLP compared to non-infected or NAP-treated cells as controls. Both viable *Mycoplasma synoviae* cells and MDLP induced elevated levels of NF-κB transcripts in HD11 cells after 1 h and 6 h (2.3- and 3.6-fold, *p* < 0.01 for *Mycoplasma synoviae*, and 3- and 3.4-fold, *p* < 0.001 for MDLP, respectively), whereas non-stimulated or NAP stimulated cells did not induce NF-κB transcription (Figure 3). The expression of *NF-κB* mRNA was inhibited after 24 h (Figure 3).

**Figure 4:**
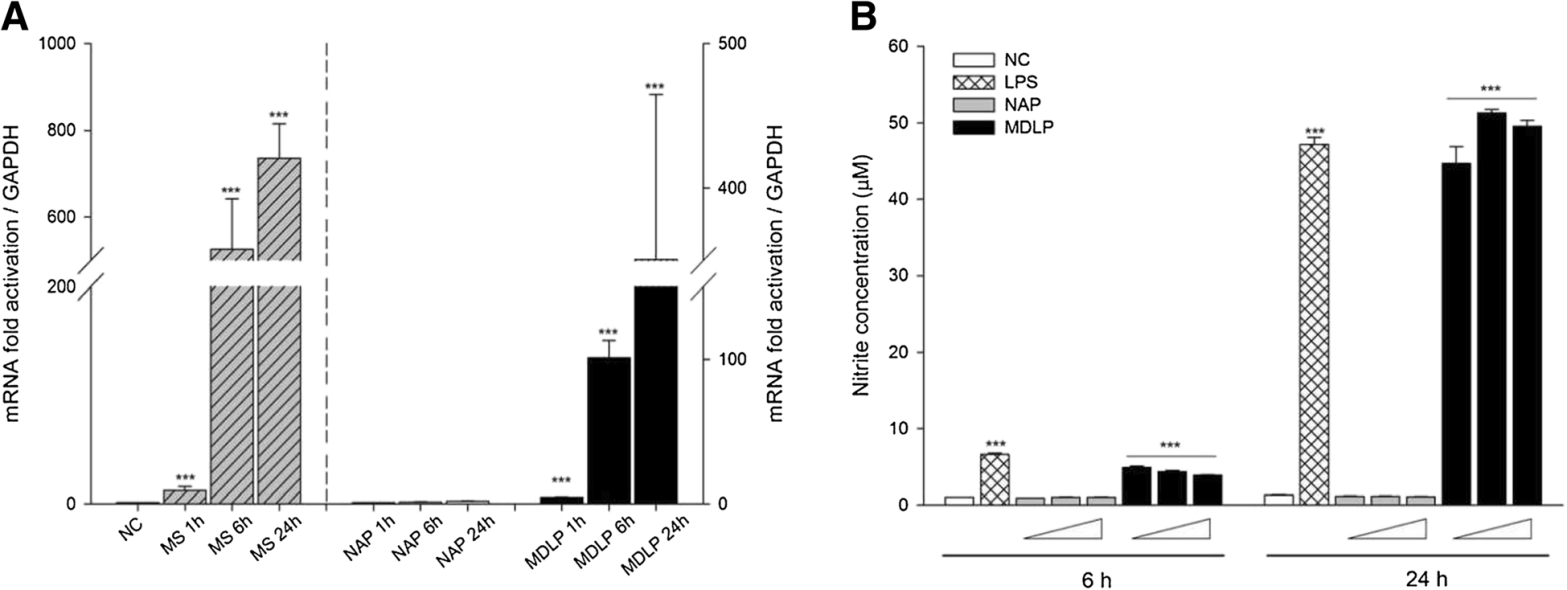
***Mycoplasma synoviae *derived diacylated lipopeptide induces *iNOS *expression and NO production. A)** Expression of the avian *iNOS* gene after infection of cells with *Mycoplasma synoviae* or treatment with diacylated lipopeptide. HD11 cells were infected with *Mycoplasma synoviae* (MS) or treated with 1 μM non-acylated peptide (NAP) or diacylated lipopeptide (MDLP) for 1 h, 6 h or 24 h and mRNA expression was analyzed by RT-qPCR. NC represents non-infected cells. Bars show mean ± S.E. (*n* = 4); ***, *p* < 0.001 compared to non-treated cells for *Mycoplasma synoviae* or NAP-treated cells at each time point, respectively. **B)** MDLP induces NO production in macrophages. HD11 cells were or treated with 0.5, 1 or 1.5 μM non-acylated peptide (NAP) or diacylated lipopeptide (MDLP) for 6 h or 24 h, and production of nitrite was measured by Griess assay. NC represents non-treated cells. LPS treated cells were used as positive control. Triangles represent increasing concentrations of NAP or MDLP. Bars show mean ± S.E. (*n* = 4); ***, *p* < 0.001 compared to non-treated cells for LPS or NAP-treated cells at concentration, respectively.

Increased *NF-κB* mRNA expression also corresponded to an increased innate immune response, as monitored by changes in *iNOS* mRNA level and its associated NO production as a functional measure of TLR15 activation. HD11 cells were infected with *Mycoplasma synoviae* or treated with 1 μM MDLP or NAP for 1 h, 6 h and 24 h, and *iNOS* mRNA levels were measured by RT-qPCR. The infection of cells with *Mycoplasma synoviae* or treatment with MDLP resulted in a significant increase in *iNOS* mRNA expression after 6 h and 24 h (Figure 4A). The treatment of cells with NAP did not induce *iNOS* expression at any time point (Figure 4A). We next investigated if elevated *iNOS* mRNA levels correlate with the production of NO in HD11 cell culture supernatants after stimulation of the cells with increasing amount of MDLP or NAP. LPS was used as a positive control. As shown in Figure 4B, treatment of cells with MDLP or LPS indeed resulted in NO production after 6 h and 24 h, whereas NAP did not stimulate NO production. In addition, MDLP induced NO at similar levels compared to LPS (Figure 4B). MDLP was also able to induce the secretion of interleukin 1β (IL-1β) and interleukin 6 (IL-6) (data not shown).

### MDLP-induced nitric oxide production is mediated via TLR15

**Figure 5:**
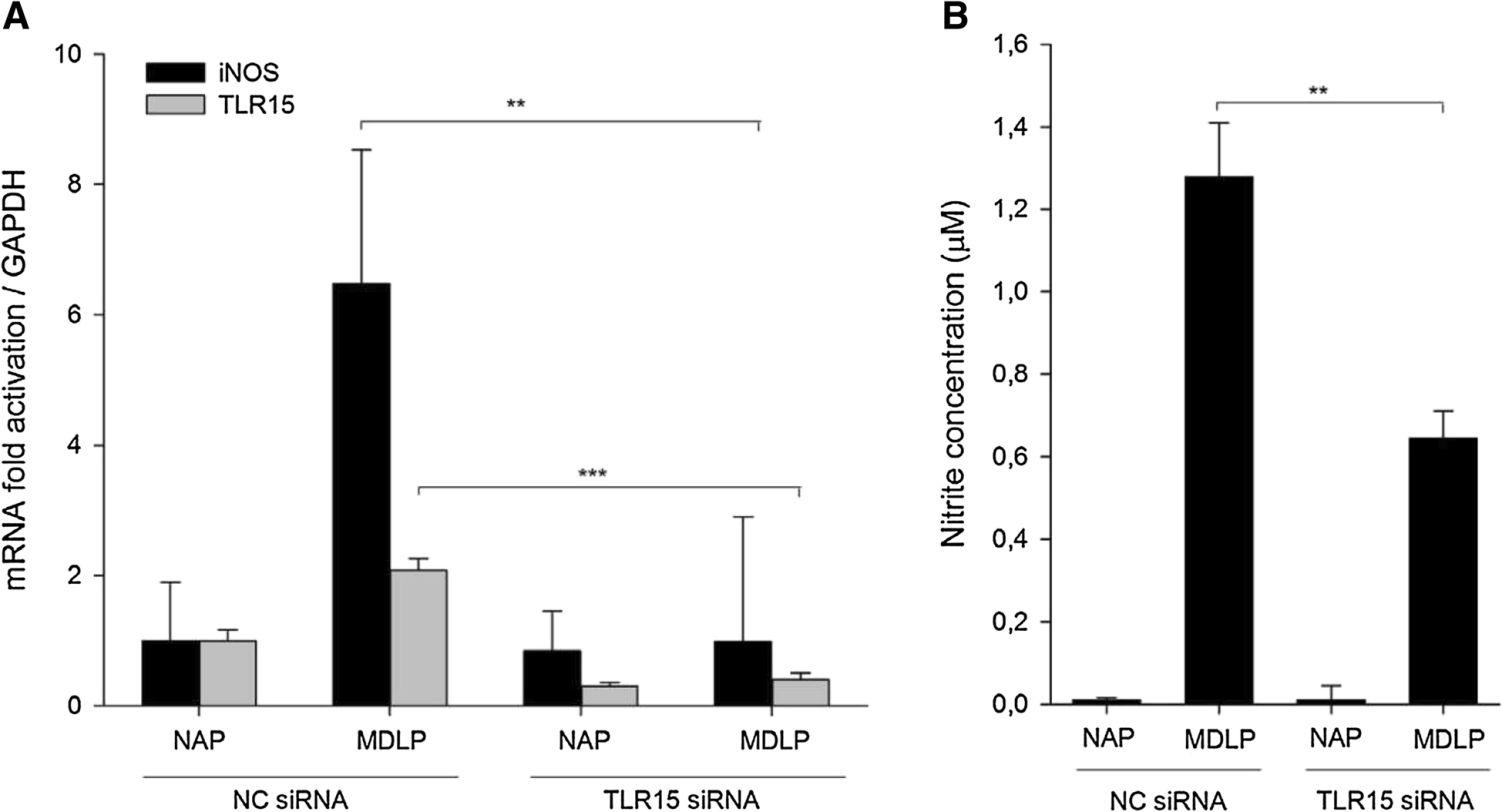
**Silencing of TLR15 decreases *iNOS *gene expression and NO production in MDLP stimulated HD11 macrophages. A)** Silencing of TLR15 decreases *iNOS* gene expression. HD11 cells were transfected with siRNA specific for TLR15 (TLR15 siRNA) or scrambled negative control siRNA (NC siRNA) for 8 h and then stimulated with NAP or MDLP for additional 12 h. mRNA expression for *iNOS* and *TLR15* was determined by RT-qPCR. Bars show mean ± S.E. (*n* = 4); **, *p* < 0.01 and ***, *p* < 0.001. **B)** Silencing of TLR15 decreases MDLP-induced NO production. HD11 cells were transfected with TLR15 siRNA or NC siRNA for 8 h and then stimulated with NAP or MDLP for additional 12 h. After, production of nitrite was measured in cell culture supernatants by Griess assay. Bars show mean ± S.E. (*n* = 4); ** *p* < 0.01.

To confirm the role of TLR15 in MDLP-mediated NO production, we knocked down *TLR15* expression in HD11 cells using siRNA. The expression level of *TLR15* diminished following transfection of HD11 cells with siRNA specific for chicken TLR15. Under stimulated conditions, cells transfected with TLR15 siRNA exhibited a decline in TLR15 mRNA expression of 70% (*p* < 0.001, NAP) and 80% (*p* < 0.001, MDLP) of the control values at 24 h post-transfection in NAP-treated or MDLP-treated cells, respectively (Figure 5A). The control transfection with scrambled siRNA (Figure 5A, NC siRNA) did not affect the expression levels of *TLR15*. *TLR15* mRNA expression was low in control NC siRNA cells under unstimulated conditions and significantly increased in response to MDLP stimulation (*p* < 0.001). The increase in *TLR15* mRNA expression induced by MDLP was significantly inhibited in TLR15-diminished cells as a consequence of effective interference of *TLR15* mRNA transcription by TLR15 siRNA (Figure 5A, *p* < 0.001). Transfection and knockdown efficiencies were evaluated by transfection of cells with TYE 563™ (Additional file 1A) and control HPRT-1 siRNA (Additional file 1B).

The influence of a reduction in TLR15 expression on iNOS induction by MDLP stimulation was also investigated. NC siRNA cells responded to treatment with MDLP with a significant increase in *iNOS* expression (Figure 5A, iNOS, *p* < 0.001). As anticipated, iNOS induction by MDLP stimulation was significantly inhibited in TLR15-deficient cells (*p* = 0.001, Figure 5A, iNOS). Furthermore, this data was corroborated by observed changes in NO production. Following treatment with DLP, NO levels in cell supernatants were markedly increased in NC siRNA cells, but NO production was significantly inhibited in TLR15-diminished cells (*p* = 0.003, Figure 5B). This data further suggests that TLR15 may be essential for iNOS induction and NO production by MDLP stimulation.

### MDLP-induced NO production is inhibited by blocking TLR15

**Figure 6:**
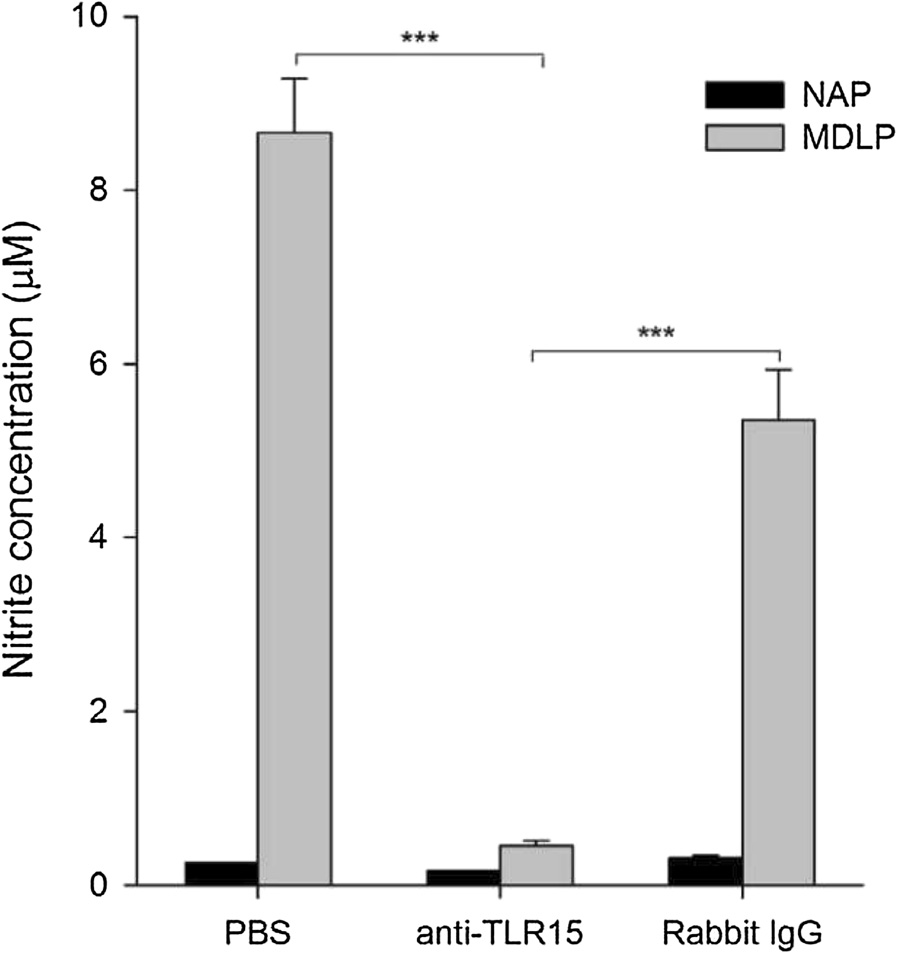
**Preincubation of HD11 macrophages with anti-TLR15 antibody decreases MDLP-stimulated NO response.** HD11 cells were preincubated with rabbit polyclonal anti-TLR15 antibody, normal rabbit serum (rabbit IgG) or phosphate buffer (PBS) for 1 h and then stimulated with NAP or MDLP for 24 h. Nitrite concentration in cell culture supernatants was measured by Griess assay. PBS-pretreated cells were used as control. Bars show mean ± S.E. (*n* = 3); ***, *p* < 0.001.

To further asses the role of TLR15 in MDLP-induced innate immune responses in chicken macrophages, we investigated the effect of blocking TLR15 with anti-TLR15 antibodies on NO production (Figure 6). Pre-incubation of HD11 cells with neutralizing rabbit polyclonal anti-TLR15 antibody for 1 h, followed by stimulation with MDLP or NAP, resulted in almost complete inhibition of NO production. Normal rabbit serum showed significantly lower inhibitory effect in comparison to TLR15 antiserum (*p* = 0.001).

## Discussion

In mammalian cells, lipopeptides mediate an innate immune response through interactions with TLR1, TLR2 and TLR6. Chickens lack TLR6 but possess two types of TLR1 and two types of TLR2 receptors that are most likely the result of gene duplication [28,29], and an additional avian specific receptor, TLR15. Therefore, this study was designed to address if TLR15 is involved in the recognition of *Mycoplasma synoviae* lipoprotein MSPB. We found that this avian pathogen, and specifically the N-terminal diacylated lipopeptide portion of MSPB (MDLP), activates the expression of TLR15 in chicken macrophages and primary chicken chondrocytes. The activation of TLR15 leads to NF-κB activation and NO production. The specificity of this interaction was confirmed by the inhibition of *iNOS* expression and NO production through the depletion of TLR15 by siRNA. Moreover, when cells were pretreated with neutralizing anti-TLR15 antibody, MDLP-mediated NO production was inhibited. We conclude that by interacting with TLR15 MDLP stimulates an innate immune response in chicken cells.

Previous studies by Lavrič et al. showed the induction of proinflamatory cytokines and chemokines, as well as iNOS, in *Mycoplasma synoviae* infected chicken macrophages [24]. Moreover, a second study showed that lipoprotein MSPB and its amino-terminal part tMSPB were responsible for a strong immune response in chicken macrophages with production of NO, IL-6 and IL-1β [7], similarly to what has been shown for other mycoplasma lipopeptides, such as MALP-2 and FSL-1 [8,30]. The N-terminal amino acid sequence of WVU 1853 strain tMSPB is CDGQTPAPEXT [7], in which two acyl chains are bound to a cysteine [8,31]. The study by Lavrič et al. also suggested that the induction of proinflamatory cytokines as well as iNOS could be a functional consequence of TLR pathway induction through *Mycoplasma synoviae* PAMPs.

In mammals, mycoplasma lipopeptides MALP-2 and FSL-1 initiate innate immune response through the TLR2/TLR6 mediated signaling pathway. However, in chickens, the signaling pathway involved in *Mycoplasma synoviae* mediated immune response has not yet been determined. We have therefore evaluated the involvement of chicken TLR2 subfamily members in the *Mycoplasma synoviae* and MSPB-derived lipopeptide, MDLP, mediated immune response. Our results show that MDLP was able to rapidly induce the expression of TLR15, whereas expression of the other two chTLR2 family members, TLR1 and TLR2, was down-regulated in response to MDLP. This result was specific for the lipid moiety since NAP did not induce any changes in the expression of chTLR2 family members.

In general, the binding of a TLR to its appropriate ligand initiates a specific signaling cascade, ultimately resulting in the activation of transcription factor NF-κB, and the expression of innate immune response genes, such as proinflamatory cytokines and iNOS. This pathway is mostly conserved in avian species [28]. In the present study, the innate immune response to MDLP in HD11 cells was monitored by the induction of iNOS and its associated NO production. We show that stimulation of chicken macrophages with *Mycoplasma synoviae* or MDLP leads to the activation of NF-κB and iNOS, which in turn leads to the production of nitric oxide. The levels of *NF-κB* and *iNOS* expression were time dependent, with *NF-κB* peaking at 6 h, followed by *iNOS* at 24 h. Participation of TLR15 in MDLP stimulation was corroborated by reducing TLR15 transcription by RNA silencing. *iNOS* expression and NO production were significantly inhibited in TLR15-diminished cells compared with control NC siRNA cells. Moreover, the MDLP-induced NO response in macrophages was abrogated by blocking antibodies specific for TLR15.

Induction of iNOS, and the resulting increase in NO production, not only has a positive role in combating infectious disease but also contributes to a number of autoimmune and inflammatory disorders. *Mycoplasma synoviae* has been associated with infectious synovitis in joints of naturally and experimentally infected chickens [3,23]. A recent study by Dušanić et al. [32] has shown that infection of primary chicken chondrocytes with *Mycoplasma synoviae* induces *NF-κB* expression and NO production. Therefore, we investigated the involvement of the chTLR2 subfamily in the recognition of *Mycoplasma synoviae* and its diacylated lipopeptide in chicken chondrocytes. Our results showed that *Mycoplasma synoviae* infection induced expression of *TLR15* mRNA in chicken chodrocytes after 24 h. The expression of *TLR1* remained unchanged, whereas the expression of *TLR2* was significantly downregulated. We also showed upregulation of *TLR15* after 6 h and 24 h of stimulation by MDLP, but not NAP, whereas the expressions of *TLR1* and *TLR2* remained unchanged. The expression of TLR2 has also been shown in human chondrocytes, where activation of the TLR2 pathway led to the production of NO, resulting in an inflammatory response [33]. In mice, the expression of TLR2 resulted in experimentally induced arthritis [34]. Taken together these findings suggest that an innate immune response, including MDLP-mediated activation of chicken chondrocytes via the TLR15 pathway, has the potential to contribute to joint inflammation in infectious synovitis.

TLR15 induction was first reported in chicken cecum following *Salmonella* infection [16]. A later study has reported the up-regulation of TLR15 expression after stimulation of cells with live and heat-killed Gram negative and Gram positive bacteria, commonly isolated from chickens, but not with equine specific pathogen *Rhodococcus equi*, therefore suggesting that TLR15 may respond specifically to avian pathogens [18]. Intriguingly, a recent study showed the ability of a yeast lysate to activate TLR15-dependent NF-kB pathways in HEK293 cells or stimulate IL-1b mRNA upregulation in chicken macrophages, which was abrogated by heat inactivation or pre-exposure of the lysate to PMSF [11]. Moreover, various TLR agonists have also been evaluated, including PAM3CSK and FSL-1 [18,21,22]. Interestingly, studies by Nerren et al. [18] and Ciraci et al. [21] showed an increase in TLR15 expression following stimulation with PAM3CSK. It has been reported previously that not only the acylation of lipopeptides, but also the peptide backbone structure is important in recognition of lipopeptides by TLR2 in mammals [35,36]. Our study differs from others in the use of avian specific *Mycoplasma* derived lipopeptide sequence to stimulate the expression of an avian specific receptor.

In conclusion, this is the first study to report the induction of TLR15 expression and activation of TLR15-mediated immune responses after stimulation with a diacylated lipopeptide derived from the avian pathogen *Mycoplasma synoviae*. These findings demonstrate MDLP as a possible ligand for TLR15. However, they do not exclude other possible ligands for TLR15. Mammalian TLR2 has a broad range of ligand specificities, and is capable of recognizing a broad repertoire of PAMPs, including several associated with Gram-positive bacteria, mycobacteria, protozoan parasites, yeast, as well as microbial lipoproteins, glycoproteins, glycolipids and non-enteric LPS [9]. TLR2 has been reported to recognize these ligands alone or in cooperation with other TLR2 family members – TLR1, TLR6 and TLR10. Further studies are needed to determine, whether TLR15 acts as homodimer or as heterodimer with other chTLRs in response to various ligands.

## Abbreviations

TLR: Toll like receptor; MDLP: *Mycoplasma synoviae*-derived diacylated lipopeptide; NAP: Non-acylated peptide; CCH: Chicken chondocyte cells; LPS: Lipopolysaccharide; PAMP: Pathogen associated molecular pattern; CpG-ODN: CpG-oligonucleotide; PAM3CSK: Tripalmitoylated lipopeptide; NO: Nitric oxide; iNOS: Inducible nitric oxide synthase; HPRT-1: Hypoxanthine phosphoribosyltransferase 1; NF-κB: Nuclear factor kappa B; GAPDH: Glyceraldehyde 3-phosphate dehydrogenase; RT-qPCR: Quantitative real time polymerase chain reaction; siRNA: Small interfering ribonucleic acid; IL: interleukin.

## Competing interests

The authors declare that they have no competing interests.

## Authors’ contributions

IO conceived the study, carried out the isolation and infection of cells, RNA isolation and subsequent gene expression analysis, siRNA and NO concentration assays, performed the statistical analysis and drafted the manuscript. KR participated in infection of cells, RNA isolation and subsequent gene expression analysis. DB carried out *Mycoplasma synoviae* cultivation and CFU determination and participated in manuscript editing. DD advised IO on RT-qPCR and performed isolation of CCH. CK participated in designing the study and manuscript editing. MN participated in designing the study, drafting the manuscript and coordination between authors. All authors read and approved the final manuscript.

## Supplementary Material

Additional file 1 **Estimation of siRNA transfection and knock-down efficiency in HD11 cells.** A) Transfection of cells with TYE 563™ as observed under fluorescent microscope. HD11 cells were transfected with 150 nM TYE 563™ oligo and its expression was observed under flourescent microscope. Transfected cells are shown in red. B) Knock down efficiency in HD11 cells transfected with HPRT-1 siRNA as measured by RT-qPCR. HD11 cells were transfected with 150 nM negative control (NC siRNA) or siRNA specific for HPRT-1 (HPRT-1 siRNA) for 24 h and mRNA expression of *HPRT-1* gene was measured by RT-qPCR. Bars show mean ± S.E. (*n* = 3); ***, *p* < 0.001. Click here for file

## References

[B1] Razin S Yogev D Naot Y Molecular biology and pathogenicity of mycoplasmas Microbiol Mol Biol Rev 1998 44 1094 1156 9841667 10.1128/mmbr.62.4.1094-1156.1998 PMC98941

[B2] Lockaby SB Hoerr FJ Lauerman LH Kleven SH Pathogenicity of Mycoplasma synoviae in broiler chickens Vet Pathol 1998 44 178 190 10.1177/030098589803500303 9598581

[B3] Narat M Bencina D Kleven SH Habe F The hemagglutination-positive phenotype of Mycoplasma synoviae induces experimental infectious synovitis in chickens more frequently than does the hemagglutination-negative phenotype Infect Immun 1998 44 6004 6009 9826385 10.1128/iai.66.12.6004-6009.1998 PMC108761

[B4] Noormohammadi AH Role of phenotypic diversity in pathogenesis of avian mycoplasmosis Avian Pathol 2007 44 439 444 10.1080/03079450701687078 17994321

[B5] Bencina D Drobnic-Valic M Horvat S Narat M Kleven SH Dovc P Molecular basis of the length variation in the N-terminal part of Mycoplasma synoviae hemagglutinin FEMS Microbiol Lett 2001 44 115 123 10.1111/j.1574-6968.2001.tb10829.x 11557149

[B6] Noormohammadi AH Markham PF Whithear KG Walker ID Gurevich VA Ley DH Browning GF Mycoplasma synoviae has two distinct phase variable major membrane antigens, one of which is a putative hemagglutinin Infect Immun 1997 44 2542 2547 9199417 10.1128/iai.65.7.2542-2547.1997 PMC175359

[B7] Lavric M Bencina D Kothlow S Kaspers B Narat M Mycoplasma synoviae lipoprotein MSPB, the N-terminal part of VlhA haemagglutinin, induces secretion of nitric oxide, IL-6 and IL-1beta in chicken macrophages Vet Microbiol 2007 44 278 287 10.1016/j.vetmic.2006.12.005 17254721

[B8] Muhlradt PF Kiess M Meyer H Sussmuth R Jung G Isolation, structure elucidation, and synthesis of a macrophage stimulatory lipopeptide from Mycoplasma fermentans acting at picomolar concentration J Exp Med 1997 44 1951 1958 10.1084/jem.185.11.1951 9166424 PMC2196331

[B9] Qureshi S Medzhitov R Toll-like receptors and their role in experimental models of microbial infection Genes Immun 2003 44 87 94 10.1038/sj.gene.6363937 12618855

[B10] Kawai T Akira S Signaling to NF-kappaB by Toll-like receptors Trends Mol Med 2007 44 460 469 10.1016/j.molmed.2007.09.002 18029230

[B11] Boyd AC Peroval MY Hammond JA Prickett MD Young JR Smith AL TLR15 is unique to avian and reptilian lineages and recognizes a yeast-derived agonist J Immunol 2012 44 4930 4938 10.4049/jimmunol.1101790 23066147

[B12] Brownlie R Allan B Avian toll-like receptors Cell Tissue Res 2011 44 121 130 10.1007/s00441-010-1026-0 20809414

[B13] Fukui A Inoue N Matsumoto M Nomura M Yamada K Matsuda Y Toyoshima K Seya T Molecular cloning and functional characterization of chicken toll-like receptors. A single chicken toll covers multiple molecular patterns J Biol Chem 2001 44 47143 47149 10.1074/jbc.M103902200 11590137

[B14] Higuchi M Matsuo A Shingai M Shida K Ishii A Funami K Suzuki Y Oshiumi H Matsumoto M Seya T Combinational recognition of bacterial lipoproteins and peptidoglycan by chicken Toll-like receptor 2 subfamily Dev Comp Immunol 2008 44 147 155 10.1016/j.dci.2007.05.003 17614130

[B15] Keestra AM de Zoete MR van Aubel RA van Putten JP The central leucine-rich repeat region of chicken TLR16 dictates unique ligand specificity and species-specific interaction with TLR2 J Immunol 2007 44 7110 7119 17513760 10.4049/jimmunol.178.11.7110

[B16] Higgs R Cormican P Cahalane S Allan B Lloyd AT Meade K James T Lynn DJ Babiuk LA O’Farrelly C Induction of a novel chicken Toll-like receptor following Salmonella enterica serovar Typhimurium infection Infect Immun 2006 44 1692 1698 10.1128/IAI.74.3.1692-1698.2006 16495540 PMC1418683

[B17] Lu Y Sarson AJ Gong J Zhou H Zhu W Kang Z Yu H Sharif S Han Y Expression profiles of genes in Toll-like receptor-mediated signaling of broilers infected with Clostridium perfringens Clin Vaccine Immunol 2009 44 1639 1647 10.1128/CVI.00254-09 19776194 PMC2772368

[B18] Nerren JR He H Genovese K Kogut MH Expression of the avian-specific toll-like receptor 15 in chicken heterophils is mediated by gram-negative and gram-positive bacteria, but not TLR agonists Vet Immunol Immunopathol 2010 44 151 156 10.1016/j.vetimm.2010.02.017 20303182

[B19] Nerren JR Swaggerty CL MacKinnon KM Genovese KJ He H Pevzner I Kogut MH Differential mRNA expression of the avian-specific toll-like receptor 15 between heterophils from Salmonella-susceptible and -resistant chickens Immunogenetics 2009 44 71 77 10.1007/s00251-008-0340-0 19002681

[B20] Shaughnessy RG Meade KG Cahalane S Allan B Reiman C Callanan JJ O’Farrelly C Innate immune gene expression differentiates the early avian intestinal response between Salmonella and Campylobacter Vet Immunol Immunopathol 2009 44 191 198 10.1016/j.vetimm.2009.06.007 19632728

[B21] Ciraci C Lamont SJ Avian-specific TLRs and downstream effector responses to CpG-induction in chicken macrophages Dev Comp Immunol 2011 44 392 398 10.1016/j.dci.2010.11.012 21095203

[B22] de Zoete MR Bouwman LI Keestra AM van Putten JPM Cleavage and activation of a Toll-like receptor by microbial proteases Proc Natl Acad Sci USA 2011 44 4968 4973 10.1073/pnas.1018135108 21383168 PMC3064367

[B23] Kleven SH Saif YM, Barnes HJ, Glisson JR, Fadly AM, McDougald LR, Swayne DE *Mycoplasma synoviae* infection Diseases of Poultry 2003 11 Ames: Iowa: State University Press 756 766

[B24] Lavric M Maughan MN Bliss TW Dohms JE Bencina D Keeler CL Jr Narat M Gene expression modulation in chicken macrophages exposed to Mycoplasma synoviae or Escherichia coli Vet Microbiol 2008 44 111 121 10.1016/j.vetmic.2007.06.011 17656046

[B25] Dusanic D Bercic RL Cizelj I Salmic S Narat M Bencina D Mycoplasma synoviae invades non-phagocytic chicken cells in vitro Vet Microbiol 2009 44 114 119 10.1016/j.vetmic.2009.02.014 19321273

[B26] Pfaffl MW A new mathematical model for relative quantification in real-time RT-PCR Nucleic Acids Res 2001 44 e45 10.1093/nar/29.9.e45 11328886 PMC55695

[B27] Willems E Leyns L Vandesompele J Standardization of real-time PCR gene expression data from independent biological replicates Anal Biochem 2008 44 127 129 10.1016/j.ab.2008.04.036 18485881

[B28] Cormican P Lloyd AT Downing T Connell SJ Bradley D O’Farrelly C The avian Toll-Like receptor pathway–subtle differences amidst general conformity Dev Comp Immunol 2009 44 967 973 10.1016/j.dci.2009.04.001 19539094

[B29] Temperley ND Berlin S Paton IR Griffin DK Burt DW Evolution of the chicken Toll-like receptor gene family: a story of gene gain and gene loss BMC Genomics 2008 44 62 10.1186/1471-2164-9-62 18241342 PMC2275738

[B30] Into T Dohkan J Inomata M Nakashima M Shibata K Matsushita K Synthesis and characterization of a dipalmitoylated lipopeptide derived from paralogous lipoproteins of Mycoplasma pneumoniae Infect Immun 2007 44 2253 2259 10.1128/IAI.00141-07 17325056 PMC1865785

[B31] Shibata K Hasebe A Into T Yamada M Watanabe T The N-terminal lipopeptide of a 44-kDa membrane-bound lipoprotein of Mycoplasma salivarium is responsible for the expression of intercellular adhesion molecule-1 on the cell surface of normal human gingival fibroblasts J Immunol 2000 44 6538 6544 11086096 10.4049/jimmunol.165.11.6538

[B32] Dusanic D Bencina D Oven I Cizelj I Bencina M Narat M Mycoplasma synoviae induces upregulation of apoptotic genes, secretion of nitric oxide and appearance of an apoptotic phenotype in infected chicken chondrocytes Vet Res 2012 44 7 10.1186/1297-9716-43-7 22280251 PMC3293721

[B33] Liu-Bryan R Pritzker K Firestein GS Terkeltaub R TLR2 signaling in chondrocytes drives calcium pyrophosphate dihydrate and monosodium urate crystal-induced nitric oxide generation J Immunol 2005 44 5016 5023 15814732 10.4049/jimmunol.174.8.5016

[B34] Joosten LA Koenders MI Smeets RL Heuvelmans-Jacobs M Helsen MM Takeda K Akira S Lubberts E van de Loo FA van den Berg WB Toll-like receptor 2 pathway drives streptococcal cell wall-induced joint inflammation: critical role of myeloid differentiation factor 88 J Immunol 2003 44 6145 6153 14634130 10.4049/jimmunol.171.11.6145

[B35] Buwitt-Beckmann U Heine H Wiesmuller KH Jung G Brock R Ulmer AJ Lipopeptide structure determines TLR2 dependent cell activation level FEBS J 2005 44 6354 6364 10.1111/j.1742-4658.2005.05029.x 16336272

[B36] Kang JY Nan X Jin MS Youn SJ Ryu YH Mah S Han SH Lee H Paik SG Lee JO Recognition of lipopeptide patterns by Toll-like receptor 2-Toll-like receptor 6 heterodimer Immunity 2009 44 873 884 10.1016/j.immuni.2009.09.018 19931471

