# Supplementary figures and images for: Diacylated lipopeptide from Mycoplasma synoviae mediates TLR15 induced innate immune responses
Source: Vet Res. 2013 Oct 17;44(1):99. doi: 10.1186/1297-9716-44-99 (PMC4014865; doi:10.1186/1297-9716-44-99)

A

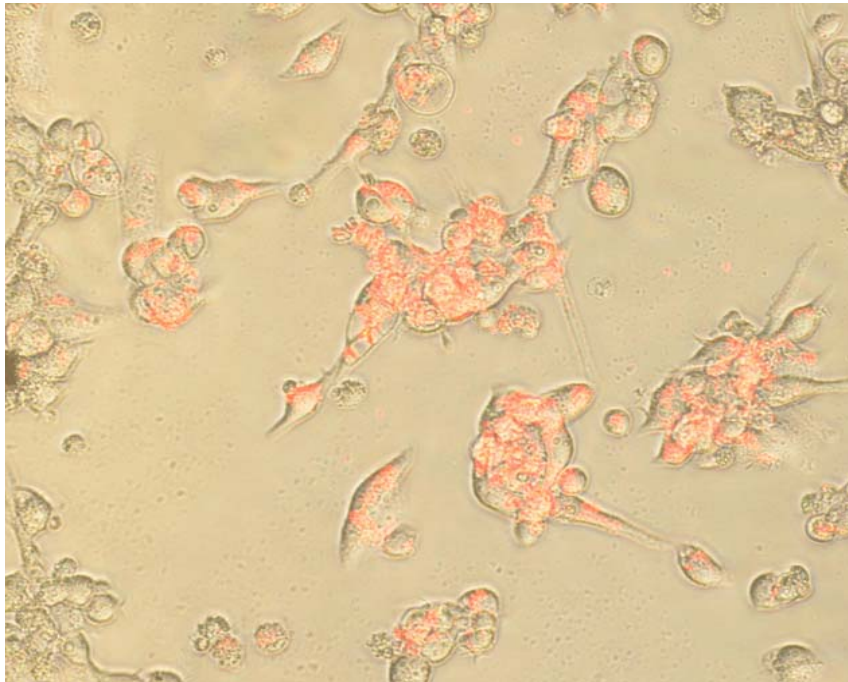

B

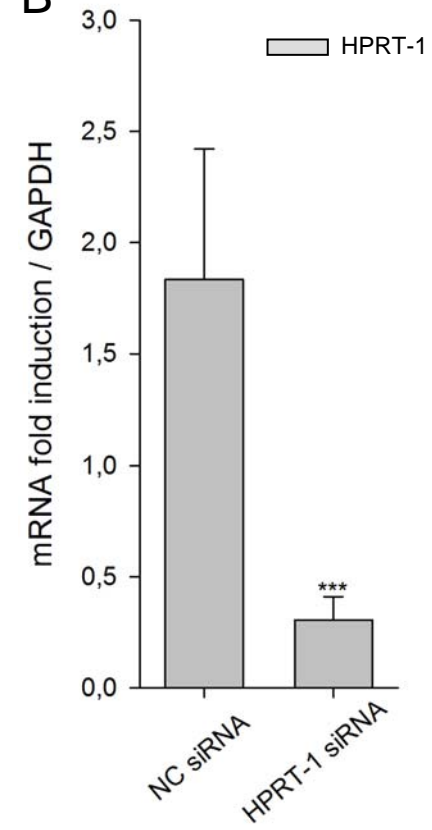

Supplement: Additional file 1 — Estimation of siRNA transfection and knock-down efficiency in HD11 cells. A) Transfection of cells with TYE 563™ as observed under fluorescent microscope. HD11 cells were transfected with 150 nM TYE 563™ oligo and its expression was observed under flourescent microscope. Transfected cells are shown in red. B) Knock down efficiency in HD11 cells transfected with HPRT-1 siRNA as measured by RT-qPCR. HD11 cells were transfected with 150 nM negative control (NC siRNA) or siRNA specific for HPRT-1 (HPRT-1 siRNA) for 24 h and mRNA expression of HPRT-1 gene was measured by RT-qPCR. Bars show mean ± S.E. (n = 3); ***, p < 0.001. [file 1297-9716-44-99-S1.pdf]
